# Virulence Factors of *Aeromonas hydrophila*: In the Wake of Reclassification

**DOI:** 10.3389/fmicb.2016.01337

**Published:** 2016-08-25

**Authors:** Cody R. Rasmussen-Ivey, Maria J. Figueras, Donald McGarey, Mark R. Liles

**Affiliations:** ^1^Department of Biological Sciences, Auburn University, Auburn, ALUSA; ^2^Departamento de Ciencias Médicas Básicas, Facultad de Medicina y Ciencias de la Salud, Institut d’Investigació Sanitària Pere Virgili, Universidad Rovira i Virgili, ReusSpain; ^3^Department of Molecular and Cellular Biology, Kennesaw State University, Kennesaw, GAUSA

**Keywords:** *Aeromonas hydrophila*, pathogenesis, comparative genomics, bacteria, phylogeny

## Abstract

The ubiquitous “jack-of-all-trades,” *Aeromonas hydrophila*, is a freshwater, Gram-negative bacterial pathogen under revision in regard to its phylogenetic and functional affiliation with other aeromonads. While virulence factors are expectedly diverse across *A. hydrophila* strains and closely related species, our mechanistic knowledge of the vast majority of these factors is based on the molecular characterization of the strains *A. hydrophila* AH-3 and SSU, which were reclassified as *A. piscicola* AH-3 in 2009 and *A. dhakensis* SSU in 2013. Individually, these reclassifications raise important questions involving the applicability of previous research on *A. hydrophila* virulence mechanisms; however, this issue is exacerbated by a lack of genomic data on other research strains. Collectively, these changes represent a fundamental gap in the literature on *A. hydrophila* and confirm the necessity of biochemical, molecular, and morphological techniques in the classification of research strains that are used as a foundation for future research. This review revisits what is known about virulence in *A. hydrophila* and the feasibility of using comparative genomics in light of this phylogenetic revision. Conflicting data between virulence factors, secretion systems, quorum sensing, and their effect on *A. hydrophila* pathogenicity appears to be an artifact of inappropriate taxonomic comparisons and/or be due to the fact that these properties are strain-specific. This review audits emerging data on dominant virulence factors that are present in both *A. dhakensis* and *A. hydrophila* in order to synthesize existing data with the aim of locating where future research is needed.

## Introduction

The ubiquitous bacterium *Aeromonas hydrophila* is a freshwater, facultatively anaerobic, chemoorganoheterotroph (Garrity et al., 2006) and the etiologic agent of disease in amphibians, birds, fishes, mammals, and reptiles, with the most common forms of disease being gastroenteritis, septicemia, and necrotizing fasciitis (Cipriano et al., 1984; Figueras et al., 2007; Monaghan et al., 2008; Janda and Abbott, 2010). Virulence in *A. hydrophila* is multifactorial, with disease resulting from the production and/or secretion of virulence factors, such as adhesins, cytotoxins, hemolysins, lipases, and proteases as well as the capacity to form biofilms, use specific metabolic pathways, and mediate virulence factor expression through quorum sensing (Allan and Stevenson, 1981; Cahill, 1990; Thornley et al., 1997; Beaz-Hidalgo and Figueras, 2013). The majority of experimental studies on identifying virulence determinants in *Aeromonas* spp. have been performed in the strain *A. hydrophila* SSU, which was later recognized to be affiliated with *A. dhakensis* on the basis of ANI and phylogeny comparisons (Grim et al., 2014). Adding confusion to this complexity, the literature on *A. hydrophila* is riddled with conflicting reports on the molecular determinants of virulence attributed to this species because of changes in classification and problems stemming from misidentification (Colston et al., 2014; Beaz-Hidalgo et al., 2015). The purpose of this review article is to provide an updated view on what is known about virulence factors in the aftermath of reclassification of *A. hydrophila* SSU.

Huys et al. (2002) recognized that some diarrheal isolates, while closely related to *A. hydrophila*, show atypical metabolic activities for urocanic acid (+), L-fucose (-), and L-arabinose (-). On these bases, these strains were classified into a subspecies known as *A. hydrophila* subsp. *dhakensis* (Huys et al., 2002). Then, in 2013, *A. hydrophila* subsp. *dhakensis* was recognized to be synonymous to *A. aquariorum* and both were combined under the name *A. dhakensis*, a species that is functionally divergent from *A. hydrophila*, based on multilocus phylogenetic analyses and phenotypic characteristics (Beaz-Hidalgo et al., 2013). Studies on the virulence factors expressed by the diarrheal isolate SSU, previously considered to be affiliated to *A. hydrophila* and now know to be *A. dhakensis*, are regarded as the seminal literature on molecular pathogenesis of *Aeromonas* (Grim et al., 2014). Given the turbulent nature of classification within *Aeromonas* spp., this review aims to clarify which virulence factors have been characterized within current members of *A. hydrophila* (**Supplementary Table S1**) by auditing the body of knowledge on the molecular understanding of these genes so that future research can progress from a more solid foundation.

## Regulation of *Aeromonas* Virulence Determinants

**FIGURE 1:**
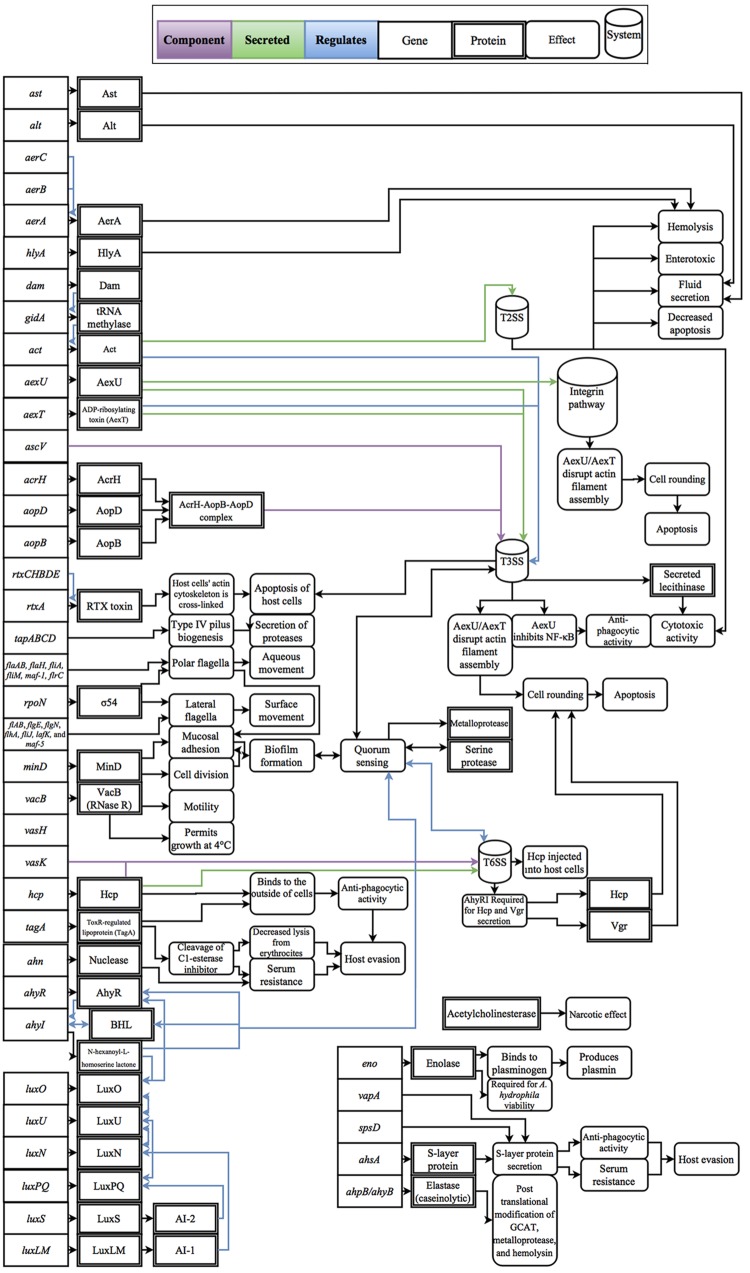
**Diagram of the gene products, molecular interactions and functions implicated in *A. hydrophila* virulence.** These interactions are based on the collective literature referenced in this manuscript.

Cascades of genetic regulation that lead to situational expression of virulence factors are known to occur in *Aeromonas* spp., but these interactions remain a relatively uncharted area of research in phylogenetically confirmed *A. hydrophila* strains. For example, outbreaks of *A. hydrophila* are generally thought to be linked with changes in host susceptibility caused by environmental changes, such as hypoxic conditions and excessive nitrite levels in farmed fish, as well as increases in temperature, which are linked with the production of virulence factors, such as cyototoxins and hemolysins (Swann and White, 1991; Mateos et al., 1993; Janda and Abbott, 2010). To exploit changes in host susceptibility due to increases in temperature, *Aeromonas* spp. virulence factors have also evolved temperature-dependent expression (Merino et al., 1992; Gonzalez-Serrano et al., 2002). For example, clinical strains of *A. hydrophila* can grow at temperatures greater than the isolate’s optimal growth temperature of 28°C (Popoff and Veron, 1976); however, when temperatures increase to 37°C, protease activity decreases and cytotoxin and hemolysin activity increases (Yu et al., 2007). In contrast, environmental isolates are well adapted to low temperatures and can grow uninhibited at temperatures as low as 4°C, a temperature that restricts growth of clinical isolates (Mateos et al., 1993). Some of the better studied regulatory effects are the linkage between quorum sensing and biofilm formation which was shown to not only mediate the expression of virulence factors, but also regulate cell density (Swift et al., 1997; Lynch et al., 2002; Janda and Abbott, 2010). In addition, while polar flagella in *A. hydrophila* are constitutively expressed, there are well-described regulators that trigger lateral flagella expression such as surface contact and viscosity (Wilhelms et al., 2011, 2013). Another class of regulatory effects includes the upregulation of virulence factors through lysogenic conversion; however, to-date no experimental data has been published on this phenomenon within *A. hydrophila*. Considering the broad effects that these regulatory factors have on disease, experimental studies that resolve these interactions are fundamental to the advancement of knowledge for the field of *A. hydrophila* as a whole. A review of known virulence factors and the respective regulatory effects that have been evaluated in *Aeromonas* spp. and are genetically present within *A. hydrophila* are presented in **Figure 1**.

## Secretion Systems

### Type II Secretion System and Effector Proteins

The widely conserved type II secretion system (T2SS) is present in all known members of *A. hydrophila* and is integral in the extracellular secretion of a wide array of virulence factors including aerolysin, amylases, DNases, and proteases (Sandkvist, 2001; Peabody et al., 2003; Tseng et al., 2009; Pang et al., 2015). In *A. dhakensis* SSU, the T2SS secretes what is perhaps the most potent virulence factor; the aerolysin-related cytotoxic enterotoxin Act (Galindo et al., 2004). While genes for this and other virulence factors that interact with the T2SS are present in current members of *A. hydrophila*, the contribution of this system to virulence remains unquantified (Cianciotto, 2005).

### Type III Secretion System and Effector Proteins

Found in higher frequency in clinical isolates than in aquatic isolates (Aguilera-Arreola et al., 2005; Pang et al., 2015), the type III secretion system (T3SS) functions as a molecular needle, injecting effector toxins into host cells (Galan and Collmer, 1999; Sha et al., 2002; Sierra et al., 2010). Although no studies have been performed in members of *A. hydrophila* with publically accessible genomic data, the T3SS has been shown in *Aeromonas* spp. to be co-regulated by contact with host cells, cytotoxic enterotoxin Act, DNA adenine methyltransferase, flagella, lipopolysaccharides, DNA methylation, temperature, calcium/magnesium levels, and quorum sensing while requiring effectors to have the appropriate secretion signal (Braun et al., 2002; Burr et al., 2003; Ebanks et al., 2006; Erova et al., 2006a,b; Vilches et al., 2009). Because of its strong association with the export of virulence factors by many *Aeromonas* spp., the experimental manipulation of genes that encode for subunits of this secretion system, which resulted in attenuation of virulence in the reclassified *A. piscicola* AH-3 (formerly *A. hydrophila*), may also result in the attenuation of *A. hydrophila* (Vilches et al., 2004; Sha et al., 2005; Vilches et al., 2008; Beaz-Hidalgo et al., 2009; Vilches et al., 2009; Khajanchi et al., 2010). At the same time, genetic heterogeneity may prevent the translation of this research. For example, calcium chelation promotes T3SS/AexT expression in *A. piscicola* AH-3 and in *A. salmonicida* JF2267, but these effects are absent in *A. salmonicida* A229, *A. salmonicida* A449 and in *A. dhakensis* SSU (Burr et al., 2003; Ebanks et al., 2006; Vilches et al., 2008, 2009). On a molecular level, *A. salmonicida* JF2267 was shown to lose its plasmid, which contains the T3SS genes, at 25°C whereas *A. salmonicida* A449 conversely increases transcription of T3SS genes between 25 and 28°C (Ebanks et al., 2006). Therefore, while the same system appears, they are different on a procedural level.

Within *A. hydrophila*, numerous studies linked the T3SS and its effector proteins with virulence. In *A. hydrophila* AH-1, an isolate of blue gourami (*Trichopodus trichopterus*) with publically available nucleotide data (whole genome is not available), insertional mutagenesis of *aopB* (T3SS translocator) and *aopD* (integral T3SS transmembrane component) causes a reduction in cytotoxicity and an increase in phagocytosis because the T3SS is no longer able to translocate effector proteins (Yu et al., 2004). Similarly, in *A. dhakensis* SSU, T3SS genes have been linked with virulence that include the T3SS-associated exoenzyme effector (AexU), which increases host evasion, degrades host actin, and is independently lethal (Sierra et al., 2010; Grim et al., 2013). AcrH is a chaperone that complexes with AopB and AopD (Tan et al., 2009); *acrH* mutants are predicted to have attenuated virulence. Contextually, the *aexU* and *acrH* genes are present in a minority of *A. hydrophila* and no experimental studies have been performed to establish their respective roles in virulence. While no experimental manipulations were performed, a subsequent study compared clinical and environmental isolates of *A. hydrophila*, showing that T3SS structural genes *aopB* and *ascV* are most abundant in *A. hydrophila* disease isolates (Carvalho-Castro et al., 2010), a link with virulence that is supported by the attenuation of virulence in *A. piscicola* AH-3 *ascV* mutants (Vilches et al., 2004). Collectively, these results appear to indicate that the T3SS is a strong contributing factor for virulence of *Aeromonas* spp. However, genomic analyses of pathogenic *A. hydrophila* isolates indicate that alternate secretory mechanisms may also be critical for pathogenesis given that hypervirulent isolates of *A. hydrophila* that infect farmed fish lack T3SS core components (Hossain et al., 2013; Pang et al., 2015).

Previously described in *A. salmonicida* (Braun et al., 2002), the ADP-ribosylating toxin AexT is present in ~90% of *Aeromonas* spp. that have a T3SS and when this T3SS effector is abrogated in *A. piscicola* AH-3, a slight attenuation of virulence has been observed based on virulence assays for cytotoxicity and phagocytosis as well as fish and mice challenges (Vilches et al., 2008). The *aexT*-like gene *aexU* shows a stronger contribution to virulence, with *aexU* mutants having an LD_50_ of 60% using 2–3 times the dose of wild-type *A. dhakensis* SSU (Sha et al., 2007; Sierra et al., 2007; Vilches et al., 2008).

### The Type VI Secretion System and Effector Proteins

The type VI secretion system (T6SS) functions analogously to a phage tail, allowing injection of virulence factors into host cells via valine glycine repeat G (VrgG) proteins and hemolysin-coregulated protein (Hcp), which functions as an antimicrobial pore-forming protein when secreted or as a structural protein (Bingle et al., 2008). In *A. dhakensis* SSU, the transcriptional regulator VasH and the helical transmembrane protein VasK are linked with secretion of Hcp, with *vasH* and *vasK* mutants resulting in decreased anti-phagocytic activity and attenuated virulence in a septicemic mouse model which serves as a line of evidence that the T6SS is involved in the manifestation of disease (Suarez et al., 2008), but similar to the disparate results of the T3SS, the T6SS is not obligatory for *A. hydrophila* virulence. For example, some members of the newly described hypervirulent *A. hydrophila* pathotype of freshwater fishes have a complete T6SS while others retain only 4/13 core components (Pang et al., 2015; Rasmussen-Ivey et al., 2016). With a distribution in 26 out of 37 strains listed as *A. hydrophila* in GenBank, the T6SS’s role in virulence may be specific to the mode of infection with bacteria that contain a complete T6SS having greater antimicrobial activity, but at the cost of stimulating host defenses. In other bacteria, the T6SS also plays a role in biofilm formation, and evasion of the host immune system, but future research is needed to assess the role(s) of the T6SS within *A. hydrophila*.

## Biofilm Formation

Biofilms provide bacteria with resistance to antimicrobial agents and host defenses (Costerton et al., 1995; Lynch et al., 2002). *Aeromonas* spp. evolved multiple regulatory mechanisms for biofilm formation that are intimately linked with the production of virulence factors. The quorum sensing response regulator of the reclassified isolate *A. piscicola* A1 (formerly *A. hydrophila*), *ahyRI*, produces LuxRI homologs, *N*-(butanoyl)-L-homoserine lactones (BHL), and *N*-hexanoyl-L-homoserine lactones (AHL); autoinducers that regulate cell division (Swift et al., 1997). In *A. dhakensis* SSU Δ*ahyRI* mutants, T6SS effectors Hcp and Vgr are unable to be secreted which results in decreased biofilm formation (Khajanchi et al., 2009). Interestingly, some strains transcribe *ahyRI* (e.g., *A. hydrophila* ATCC 7966), but lack AHL/BHLs, which may indicate an alternate function of *ahyRI* that has yet to be described (dos Reis Ponce-Rossi et al., 2016). Similarly, the recently characterized autoregulatory two-component signal transduction system QseBC is a widely conserved system within *A. hydrophila* and was first described in *A. dhakensis* SSU, as mutants with an inactive response regulator (QseB) have reduced swimming and swarming motility, form thicker biofilms, and secrete fewer virulence factors, which leads to attenuation of virulence. When the gene *aha0701h* is overexpressed in Δ*qseB* mutants, biofilm formation decreases, presumably due to dysregulation of genes *fleN* (regulates flagellar number) and *vpsT* (transcriptional response regulator; Khajanchi et al., 2012; Kozlova et al., 2012).

## Flagella and Pili

*A. hydrophila* isolates produce lateral flagella for surface movement/swarming and polar flagella for movement in suspension. Polar flagella production has been studied within *A. piscicola* AH-3, with mutations in *flaAB*, *flaH*, *fliA*, *fliM*, *maf-1*, and *flrC* abolishing production of polar flagella and resulting in decreased adherence and biofilm formation (Canals et al., 2006). Considering that flagellar glycosylation was shown to be linked with the ability to form biofilms as well as adhere to Hep-2 cells, it is important to mention that there are notable differences within *Aeromonas* species. In addition to having only a single lateral flagellin, polar and lateral flagella are glycosylated in *A. piscicola* AH-3 whereas *A. hydrophila* AH-1 has two lateral flagellins and only the polar flagellum is glycosylated. When pseudaminic acid biosynthesis genes *pseB* and *pseI* were mutagenized, the result was an inability to produce both polar and lateral flagella in *A. piscicola* AH-3, but only affected polar flagella production in *A. hydrophila* AH-1. Therefore, lateral flagella production was unaffected in glycosylation negative *A. hydrophila* AH-1 mutants (Fulton et al., 2015). Similarly, in the diseased eel isolate *A. hydrophila* W (no genome submitted), mutations in *flgE*, *flgN*, *flhA*, *fliJ*, *flmB*, *lafK*, and *maf-5* result in loss of lateral flagella, which causes decreased motility, biofilm formation, and mucosal adherence (Jiang et al., 2015). While polar and lateral flagella transcriptional hierarchies, regulation, and contribution to virulence are well-described in other species, as the date of this publication, no member of *A. hydrophila* with a publically accessible genome has undergone genetic manipulations to evaluate the contribution of polar or lateral flagella for virulence.

The *A. hydrophila* gene cluster *tapABCD* is responsible for type IV pilus biogenesis and is an integral part of the extracellular secretory pathway. To test for function, the *A. hydrophila* Ah65 (genome unavailable) gene *tapD* gene was used to successfully complement a strain of *Pseudomonas aeruginosa* that lacks PilD (an ortholog of TapD; Pepe et al., 1996). Another type IV pilus is the bundle-forming pilus, which is encoded by *bfp* and acts an important internal colonization factor for multiple species of *Aeromonas* (*A. hydrophila* Ah65 was observed expressing both *bfp* and *tap*; Barnett et al., 1997; Kirov et al., 2000). Taken with the observation that TapD is required for secretion of virulence factors, such as aerolysin and proteases, these genes appear to be fundamental for pathogenicity.

## Structural Proteins, Phospholipids, and Polysaccharides

Capsules, *O*-antigens, and S-layer proteins provide mechanisms to evade host defenses. Within *Aeromonas* spp., capsules also show anti-phagocytic activity, increase resistance to the complement system, and increase adherence (Martinez et al., 1995; Merino et al., 1997). *O*-antigens are a class of structurally diverse lipopolysaccharides that act as colonization factors. At 20°C *O*-antigen is produced by *A. piscicola* AH-3, but not at 37°C, resulting an *O*-antigen-deficient strains that are unable to colonize hosts and have reduced expression of T3SS components (Merino et al., 1996; Vilches et al., 2009). Across *A. hydrophila*, eight distinct *O*-antigen gene clusters are present, with all epidemic strains isolated from channel catfish (*Ictalurus punctatus*) sharing a homologous *O*-antigen gene cluster (Hossain, 2012). In *A. hydrophila* TF7 (genomic data unavailable), the S-layer protein gene (*ahsA*) encodes an external paracrystalline layer that is lost upon insertional mutagenesis of *spsD* (S-protein secretion; Thomas and Trust, 1995). Another study of S-layer proteins in five pathogenic human and eel isolates of *A. hydrophila* (A19, AH290, E37, E40, and TW1; genomic data unavailable) shows that serogroups of *A. hydrophila* other than *O*:11 contain S-layer proteins *O*:14 and *O*:81 (Esteve et al., 2004).

## Hemolysins

Hemolysins are a diverse group of multifunctional enzymes that play a central role in *A. hydrophila* pathogenesis (Wadstrom et al., 1976; Asao et al., 1984). The extracellular heat-labile hemolysin (AHH1) is the most abundant of several widely distributed hemolysins (AerA, AHH1, AhyA, and Asa1), with the most cytotoxic genotype being a synergistic combination of *aerA* and *ahh1* (Hirono and Aoki, 1991; Wang et al., 2003). In *A. media* A6 (formerly *A. hydrophila*) Aerolysin A (*aerA*) and Hemolysin A (*hlyA*) comprise another two-component hemolytic system in which virulence is attenuated only when both *hlyA* and *aerA* activity is abolished (Wong et al., 1998). In *A. dhakensis* SSU, the iron dependent, *fur* and *gidA*-regulated, enterotoxin Act is the most cytotoxic virulence factor of and a core gene within *A. hydrophila*, with studies in *A. dhakensis* SSU demonstrating that Act induces multiple effects including hemolytic, cytotonic, and cytotoxic activities, but unlike other virulence factors exported via the T3SS or T6SS, Act is exported through the T2SS (Chopra et al., 2000; Sha et al., 2001, 2004, 2005; Fadl et al., 2006; Erova et al., 2012).

## Collagenase, Serine Protease, Metalloprotease, Enolase, and Lipase

*A. hydrophila* spp. express diverse degradative enzymes that can contribute to virulence including collagenase, elastase, enolase, lipase, metalloprotease, and serine protease. *A. piscicola* AH-3 contains a collagenase, which has sequence similarity to the open reading frame AHA_0517 of *A. hydrophila* ATCC 7966^T^, and was shown to be cytotoxic to Vero cells, with loss of this enzyme resulting in a 5–15% increase in cell viability; however, this mutation did not result in complete reduction of cytotoxicity (Duarte et al., 2015). The *ahpAB* genes of *A. hydrophila* AG2 (genomic data unavailable) produce potent virulence factors: an extracellular protease that is not essential for virulence, but is present in the most virulent pathotypes (AerA^+^Alt^+^Ahp^+^) along with a secreted elastase with caseinolytic and elastolytic activity that correlates with an LD_50_ 100 times more virulent than AhpB^-^ mutants when assayed in rainbow trout (*Oncorhynchus mykiss*; Rivero et al., 1991; Cascon et al., 2000; Li et al., 2011). Another extracellular protease (*epr*) was discovered in the soft-shell turtle isolate *A. hydrophila* AH1 and found to be present in the most common pathotype in diseased fishes (Aer^+^Alt^+^Act^+^EprCAI^+^Ahp^+^; Chang et al., 1997; Hu et al., 2012). In the rainbow trout isolate *A. hydrophila* B32, a novel serine protease (*ser*) was found that exhibits cytotoxic properties and is thermostable, both of which are characteristics that differentiate this protease from known *A. hydrophila* α-hemolysins and β-hemolysins (Rodriguez et al., 1992). While four times less active than serine protease, the virulent *A. hydrophila* EO63 (genomic data unavailable) was shown to produce a thermostable metalloprotease with enzymatic activity on casein and elastin, an optimal pH of 8.0, and an LD_50_ of 3.5 μg/g (Esteve and Birbeck, 2004). Enolase, a secreted and surface-expressed glycolytic enzyme, was identified as a virulence factor in *A. dhakensis* SSU, based on binding to human plasminogen which leads to production of plasmin (degrades blood plasma proteins), with previous reports showing that enolase functions as a heat-shock protein and a regulator of transcription by binding host chromatin/cytoskeletal structures as well as being necessary for viability (Sha et al., 2009).

In general, lipases have diverse functions, but are linked with virulence in numerous pathogens (Stehr et al., 2003). An extracellular lipase (EC3.1.1.3) is produced by *A. piscicola* AH-3 (formerly *A. hydrophila*); however, the link between virulence and this gene is speculative in *A. hydrophila* (Anguita et al., 1993). Conversely, the heat-labile lipase Alt and the heat-stable lipase Ast are important cytotonic enterotoxins in the pathogenicity of *A. dhakensis* SSU, with both being able to cause significant fluid secretion, with only the previously described cytotoxic enterotoxin Act having a greater effect on fluid secretion (Sha et al., 2002; Li et al., 2011). Based on comparative genomics, Alt and Ast are core elements of *A. hydrophila*; however, no experiments have been performed within existing members of this species to characterize these toxins. Two additional lipases, phospholipase A1 (*pla*) and phospholipase C (*plc*), were explored in *A. piscicola* AH-3, with the finding that *pla* lacks a significant effect on virulence while *plc* (lecithinase) was cytotoxic and has LD_50_ values 10 times more virulent than *plc*-deficient mutants (Merino et al., 1999).

## Other Virulence Factors

The range of virulence factors encoded by *A. hydrophila* includes adherence proteins, catalysts, nucleases, and toxins that may be expressed differently depending upon the respective environment. The role of the adhesin *minD* in virulence is its ability to mediate mucosal adherence, increase biofilm formation, and facilitate cell division as well as motility (Huang et al., 2015). The enzyme 5-enolpyruvylshikimate 3-phosphate synthase (encoded by *aroA*) is required for folate availability and contributes to *A. hydrophila* AG2 (genomic data unavailable) viability in intraperitoneally injected rainbow trout, with *aroA* mutants no longer recoverable from fish internal organs because environmentally derived folate is scarce (Hernanz Moral et al., 1998). Another element of host evasion is the nuclease encoded by the *ahn* gene of *A. hydrophila* J-1 which shows no significant change in hemolytic activity or growth *in vitro*; however, when Δ*ahn* mutants are introduced into fish and mice models, virulence is attenuated (Ji et al., 2015). Another conserved gene of *A. hydrophila* that has only been characterized in *A. dhakensis* SSU is *vacB*, which encodes RNase R; an exoribonuclease with multiple functions that include permitting growth at 4°C (cold-shock protein) and supporting motility. Isogenic mutants of RNase R show a 70% attenuation in virulence (Erova et al., 2008).

Another virulence factor that is shown to increase host evasion for *A. dhakensis* SSU is the pore-forming RTX toxin RtxA that requires contact with host cells and is regulated by the *rtxACHBDE* operon so that production of RtxA coincides with regulation of other cytotoxins, such as aerolysins and hemolysins and acts to covalently cross-link host cytoskeletal-actin, resulting in host cells having a rounded phenotype that leads to apoptosis (McCoy et al., 2010; Suarez et al., 2012). Another conserved virulence factor across all *A. hydrophila* strains in GenBank is the ToxR-regulated lipoprotein (TagA) of *A. dhakensis* SSU, which cleaves the complement C1-esterase inhibitor, thereby increasing serum resistance and decreasing erythrocyte lysis (Pillai et al., 2006).

## The Role of Horizontal Genetic Transfer in Virulence

The introduction of virulence factors as well as their effects on the alternate regulation within *Aeromonas* spp. is a recurring theme of crucial importance, yet these elements remain understudied. Plasmids are a confirmed source of multidrug resistance in *Aeromonas* spp. and have been shown to have the potential to be conjugally transferred between known human pathogens, such as *Acinetobacter baumannii* AYE and *A. hydrophila* (Del Castillo et al., 2013). In addition to being used as a “molecular map” to identify ancestral lineages, prophage that contain putative *cis*-acting elements and *trans*-acting factors were found to be conserved within hypervirulent strains of *A. hydrophila* which strongly implies that the differential regulation of virulence factors (and therefore the dramatic increase in virulence) may be caused by the lysogenic conversion of this conserved *A. hydrophila* lineage by these mobile genetic elements (Hossain et al., 2013). Of note, while *A. dhakensis* SSU contains the majority of virulence factors that are present within confirmed members of *A. hydrophila*, all other members of *A. dhakensis* with fully sequenced genomes appear to lack these genes (**Supplementary Table S1**). Results of numerous core genome phylogenies and average nucleotide identity analyses support the grouping of *A. hydrophila* SSU within *A. dhakensis* (Colston et al., 2014; Beaz-Hidalgo et al., 2015; Rasmussen-Ivey et al., 2016), if virulence factors are introduced or controlled by mobile genetic elements, then taxonomy and functionality demand separate analyses.

## Discussion

In *Aeromonas* spp., as with all pathogens, disease is the result of complex molecular interactions between bacterium, environment, and host; however, the literature on *A. hydrophila* remains limited by the lack of experimental data on validated members of *A. hydrophila*. While there numerous virulence factors shared between members of *A. hydrophila*, *A. dhakensis* SSU, and *A. piscicola* AH-3 there are also key examples in the literature that show conflicting data between virulence factors, secretion systems, quorum sensing, and their effect on pathogenicity. This inconsistency is illustrated by the highly virulent catfish isolate *A. hydrophila* ML09-119 that acts as a primary pathogen when other members of this species act as secondary pathogens (Griffin et al., 2013; Zhang et al., 2013). With that in mind, the thorough research conducted on *A. dhakensis* SSU by researchers, such as Dr. Ashok Chopra, still holds relevance for *A. hydrophila*, but future research should be mindful of the phylogenetic reclassification for strains AH-3 and SSU and that there may be significant differences in the molecular determinants of virulence for *A. hydrophila.*

To compare isolates of *A. hydrophila*, biochemical, morphological, and molecular techniques are required (Abbott et al., 2003; Martinez-Murcia et al., 2011; Beaz-Hidalgo et al., 2015). As of 2016, few strains exist that have enough supporting data to facilitate comparative studies. There are many sources of uncertainty when comparing *A. hydrophila* strains, including genetic heterogeneity, the lack of natural models of infection, and reclassification of bacterial strains as new data emerges. Future research should aim to couple typing techniques (e.g., genome sequencing) with experimental data on virulence determinants so that there is a clear phylogenetic context for these studies.

When considering known virulence factors, the definitive biological separation of *A*. *hydrophila*, *A. piscicola* AH-3, and *A. dhakensis* SSU has yet to be established. In *A. hydrophila* as in these other species, disease is the result of a molecular symphony, with each virulence factor contributing to a cumulative effect (**Figure 1**). Research studying novel virulence factors and regulatory effects will help unveil the determinants that allow for infection and what differentiates *A*. *hydrophila* from other aeromonads. To better understand *A. hydrophila* pathogenesis it is imperative that future research develops natural models of infection, assesses the role of mobile genetic elements in virulence, and quantifies the interplay between virulence factors and host response in concert with molecular genetic approaches.

## Author Contributions

CR-I, MF, DM, and ML all contributed to the conception, writing and editing of this manuscript.

## Conflict of Interest Statement

The authors declare that the research was conducted in the absence of any commercial or financial relationships that could be construed as a potential conflict of interest.

## Supplementary Material

TABLE S1 **Gene products implicated in *A. hydrophila* virulence and the relative coverage and identity of strains listed in GenBank against the strain from which the gene product was characterized**.
